# Assessing Restricted and Repetitive Behaviours in Online-Sampled Autistic and Non-autistic Individuals: Factor Structure of the Repetitive Behaviours Questionnaire for Adults (RBQ-2A)

**DOI:** 10.1007/s10803-023-05977-w

**Published:** 2023-04-13

**Authors:** Jack D. Brett, Brooke Peden, David A. Preece, Andrew Whitehouse, Rodrigo Becerra, Murray T. Maybery

**Affiliations:** 1 The University of Western Australia, Perth, Australia; 2 Curtin University, School of Psychology, Perth, Australia; 3 Telethon Kids Institute, The University of Western Australia, Perth, Australia; 4 Cooperative Research Centre for Living With Autism (Autism CRC), Perth, Australia

**Keywords:** Autism, Restricted and repetitive behaviours, Questionnaire, Exploratory structural equation modelling, Confirmatory factor analysis, Adult

## Abstract

The Repetitive Behaviours Questionnaire for Adults (RBQ-2A) measures two factors of restricted and repetitive behaviours (RRBs) associated with autism. However, the Diagnostic and Statistical Manual of Mental Disorders (DSM-5) provides four criteria for RRBs: repetitive motor behaviours, insistence on sameness, restricted interests, and interest in sensory aspects of the environment (or atypical sensitivity). The current paper aimed to examine whether the RBQ-2A is a psychometrically sound measure of these four factors. Study 1 had university students (*N* = 368) complete the RBQ-2A and other related measures online and revealed that the RBQ-2A can assess the factors highlighted in the DSM-5 and that these four factors comprise a general RRB construct. Study 2 had individuals disclosing a diagnosis of autism (*N* = 283) complete the RBQ-2A and other related measures online and supported that this four-factor structure provided good psychometric properties. While the current paper provides findings for an online autistic population, further research is needed to generalize these findings to autistic individuals less likely to partake in online studies (e.g., those with intellectual or language disabilities). Overall, the results suggest that the RBQ-2A reliably and validly assesses RRBs.

**Supplementary Information:**

The online version contains supplementary material available at 10.1007/s10803-023-05977-w.

Autism Spectrum Disorder (autism) is characterised by differences within social and non-social dimensions of human behaviour, i.e., atypical social behaviours and the presence of restricted and repetitive behaviours (RRBs; Fletcher-Watson & Happé, 2019). As defined in the Diagnostic and Statistical Manual of Mental Disorders (DSM-5), RRBs encompass a wide array of autistic-like behaviours: (1) repetitive motor movements, (2) insistence on sameness, (3) restricted interests, and (4) interest in sensory aspects of the environment (or hyper-/hypo-sensitivity to sensory input; American Psychiatric Association [APA], 2013).

While conceptualisations of autism propose a general RRB construct (e.g., English et al., 2021; Shuster et al., 2014; Warrier et al., 2019), this construct comprises multiple behaviours varying across developmental and clinical variables (Uljarević et al., 2022). For example, Grove et al. (2021) found a four-factor structure for RRBs containing repetitive motor movements, insistence on sameness, restricted interest, and sensory sensitivity, mapping neatly to the given criteria within the DSM-5 (APA, 2013). While these behaviours are distinct and varied (Uljarević et al., 2022), they may occur together along a spectrum (Zheng et al., 2019). However, to our knowledge, no study has directly tested whether a higher-order/general RRB factor exists.

The Repetitive Behaviours Questionnaire (RBQ-2) is a commonly used parental-report measure of RRBs. Factor analyses of the RBQ-2 have shown evidence of a two-factor solution (Lidstone et al., 2014) comprising a repetitive motor/sensory behaviours factor and an insistence on sameness factor, and a four-factor solution similar to Grove et al., (2021; Leekam et al., 2007). Leekam et al. (2007) suggested that the four-factor solution allows for the best interpretation. The RBQ-2 has been adapted as a self-report questionnaire for adults (RBQ-2A; Barrett et al., 2015). While the RBQ-2A is a derivative of the RBQ, studies have only assessed the RBQ-2A with a two-factor solution, incorporating repetitive motor/sensory behaviours and insistence on sameness (Barrett et al., 2015, 2018). To our knowledge, the RBQ-2A has not been explored as a potential measure of four factors similar to the RBQ-2, which may map onto the DSM-5 RRB criteria (APA, 2013).

The two validation studies of the RBQ-2A (Barrett et al., 2015, 2018) examined the RBQ-2A’s psychometric properties in university students and adults with and without autism. Their findings suggested that the measure contained two factors based on principal components analysis with parallel analysis. Parallel analysis (Horn, 1965) is a valuable tool for accurately making dimensionality decisions (e.g., Henson & Roberts, 2006). However, a parallel analysis relies on product-moment correlation matrices, which creates biases when using ordered categorical variables (as for items of the RBQ-2A). These biases lead to underestimated factor loadings and standard errors (Muthén & Kaplan, 1992) and compromise parallel analysis results (Lubbe, 2019). As such, past studies (e.g., Barrett et al., 2015; Lidstone et al., 2014) may have found only two factors due to this biased approach. Direct model testing a comparison of the two and four-factor solutions (i.e., within a confirmatory factor analysis [CFA] or the exploratory structural equation model [ESEM] framework) may therefore be more appropriate.

Additionally, the two validation studies (Barrett et al., 2015, 2018) excluded the last item (i.e., “What sort of activity will you choose if you are left to occupy yourself?”) from factor analyses as its response scale differs from the other items (i.e., (1) “A range of different and flexible self-chosen activities”; (2) “Some varied and flexible interests but commonly choose the same activities”; (3) “Almost always choose from a restricted range of repetitive activities”). While this item does have a different response scale, the item responses are on an ordinal categorical scale (i.e., increase in the level of restricted activities) and can still be included within a factor analysis (Jöreskog & Moustaki, 2001). Furthermore, this item is used in the standard scoring procedure when deriving a total score for RRBs (e.g., Brett & Maybery, 2022; Hwang et al., 2020). Therefore, it is essential to investigate the appropriateness of its inclusion.

The present paper reports the results of two studies investigating the factor structure of the RBQ-2A within a university and an online autistic sample. Study 1, using a university sample, aimed to establish the factor structure of the RBQ-2A and provided convergent and discriminant validity. To avoid the potential biases of parallel analysis with ordinal categorical variables, ESEMs were used to determine the appropriate number of factors, using model fit indices and interpretability (Finch, 2020). CFAs were then conducted to see if a new factor structure showed a better model fit than the original structure (i.e., the two-factor model from Barrett et al., 2015). Furthermore, a hierarchical CFA was investigated to assess whether RRBs may represent a single multi-dimensional construct. Lastly, convergent validity was assessed with other measures of social and non-social autistic traits.

Study 2 aimed to replicate this factor structure within an autistic sample. In addition, measurement invariance testing was conducted to provide evidence for whether the RBQ-2A produced psychometric biases when comparing autistic and non-autistic individuals. Lastly, further convergent and discriminant validity was assessed with another measure of autistic traits.

## Study 1

### Method

#### Participants and Procedure

There were 368 participants^1^ (33% male, 67% female) recruited from an undergraduate psychology university course (61% Australian, 14% British, 14% Chinese, 16% European, 4% Indian, 2% Vietnamese, 1% Indigenous and/or Torres Strait Islander^2^), with a mean age of 19.44 years (SD = 2.45; Range: 17—36). Participants completed a series of questionnaires online through Qualtrics (materials below).

#### Materials

The RBQ-2A (Barrett et al., 2015) is a 20-item self-report scale assessing RRBs in adults. The questionnaire consists of five sections, differing on the Likert scale used. In particular, two sections use a 3-point Likert scale (Sect. 1: items 1–5; Sect. 5: item 20), while the other three use a 4-point Likert scale (Sect. 2: items 6–12; Sect. 3: items 13–16; Sect. 4: items 17–19). Higher scores indicate a greater tendency for RRBs.

The Autism-Spectrum Quotient (AQ; Baron-Cohen et al., 2001) is a 50-item self-report scale assessing autistic traits in adults. We used the three-factor solution identified by Russell-Smith et al. (2011; as recommended by English et al., 2020): (1) social difficulties, (2) communication difficulties, and (3) attention to details. Participants respond on a 4-point Likert scale ranging from *Definitely Agree* (1) to *Definitely Disagree* (4). Higher scores indicate more significant difficulties in social skills or communication or greater attention to detail. In the present study, the social difficulties and attention to detail factors showed good internal consistency reliability (Cronbach’s *α* = 0.80–0.88). However, the communication difficulties factor showed poor internal consistency reliability (Cronbach’s *α* = 0.65).

The Glasgow Sensory Questionnaire (GSQ; Robertson & Simmons, 2013) is a 42-item self-report scale assessing atypical sensory responsiveness (both hyper- and hypo-sensitivity across multiple sensations) in adults. Participants respond on a 5-point Likert scale ranging from *Never* (1) to *Always* (5). Higher scores indicate greater atypical sensory responsiveness (either hyper- or hypo-sensitivity). The total score showed good internal consistency reliability in the present study (Cronbach’s *α* = 0.93).

The Intolerance of Uncertainty Scale—short form (IUS-12; Carleton et al., 2007) is a 12-item self-report scale assessing intolerance of uncertainty in adults. Participants respond on a 5-point Likert scale ranging from *not at all characteristic of me* (1) to *entirely characteristic of me* (5). Higher scores indicate greater intolerance of uncertainty. The total score showed good internal consistency reliability in the present study (Cronbach’s *α* = 0.93).

#### Statistical Analysis

All statistical analyses were conducted in Mplus 8.0, except for obtaining Cronbach’s *α,* which was done via Jamovi 2.2.5. The factor structure was assessed in two parts. Firstly, ESEMs were used to ascertain the number of factors and highlight redundant items. Secondly, a series of CFAs were used to investigate whether this factor structure showed a good model fit compared to the original structure proposed by Barrett et al. (2015) and whether a hierarchical variant is appropriate.

ESEMs (with Geomin rotation) with an increasing number of factors were conducted to determine the factor structure of the RBQ-2A. As the items from the RBQ-2A are ordinal categorical, the weighted least square mean and variance adjusted (WLSMV) estimator was used (Rhemtulla et al., 2012). The most appropriate model (i.e., the number of factors to extract) was determined based on the model fit indices and interpretability of the structure.

Finch (2020) suggested that change in model fit indices outperformed parallel analysis in determining the appropriate number of factors to extract for categorical data. As such, multiple model fit indices were used to assess model fit. Comparative fit index (CFI) and Tucker Lewis index (TLI) values ≥ 0.95 were judged to indicate good fit, as were root mean square error of approximation (RMSEA) values ≤ 0.06 (Bentler & Bonnet, 1980; Browne & Cudeck, 1992; Hu & Bentler, 1999; Marsh et al., 2004). However, changes in model fit indices were examined to determine the number of factors. For a model to show significant improvement in model fit, CFI/TLI values should increase by at least 0.010, and RMSEA values should decrease by at least 0.015 (Finch, 2020). If extracting an additional factor did not show a significant improvement in model fit, the more parsimonious model (i.e., fewer factors) would be preferred.

While changes in model fit indices can overcome some biases from parallel analysis with ordinal categorical data, some limitations exist (Clark & Bowles, 2018; Garrido et al., 2016). As such, interpretation of the factor loadings will also guide in determining the number of factors to extract. Consistent with past research on the RBQ-2A (Barrett et al., 2015, 2018), factor loadings were considered meaningful when larger than |.40|. Furthermore, Montoya and Edwards (2021) found that model fit indices can be overly sensitive to correlated residuals, thus overestimating the number of factors. As such, potential correlated residuals were modelled within the ESEMs, where appropriate.

After completing the series of ESEMs, multiple CFAs were conducted. Three models were assessed: (1) the original factor structure from Barrett et al., (2015; i.e., a repetitive motor/sensory behaviours factor and an insistence on sameness factor), (2) the model suggested by the ESEMs, and (3) a hierarchical variant of the previous model to assess whether the factors may form an overall RRB score. In addition, modification indices were examined to investigate any potentially significant cross-loadings.

Cronbach’s alpha (*α*), McDonald’s omega (*ω*), and hierarchical omega (*ω*_*H*_; using a bi-factor model; Supplementary Material, Table S4) were calculated for the RBQ-2A factors. Internal reliability coefficient values ≥ 0.70 were considered acceptable, values ≥ 0.80 were considered good, and values ≥ 0.90 were considered excellent (Groth-Marnat, 2009).

To assess convergent and discriminant validity, Pearson correlations were computed between the RBQ-2A latent factors and scales from the AQ, GSQ, and IUS-12. The latent factors of the RBQ-2A were obtained using the preferred CFA model, while the scales from the other measures were inputted as observed scores. It was expected that the RBQ-2A factors would have greater correlations with the non-social rather than the social scales of the other measures.

### Results

#### Exploratory Structural Equation Modelling

Increasing the number of factors showed significant improvements in model fit indices, with the five-factor solution showing the best model fit (Supplementary Material, Table S1). However, only items 9 and 10 meaningfully loaded onto the fifth factor, which may result from these items being parallel-worded (i.e., “Have special interest in the…”). As such, we allowed items 9 and 10 to co-vary in the four-factor model (the items had a polychoric correlation of 0.25, *p* < 0.001). This model explained 51.64% of the questionnaire’s variance, provided good model fit indices (RMSEA = 0.036, CFI = 0.989, TLI = 0.982), and showed no significant difference in fit compared to the five-factor model (ΔRMSEA = 0.008, ΔCFI = − 0.005, ΔTLI = − 0.007). In addition, the model had no significant cross-loadings and all items meaningfully loaded onto a factor (≥ 0.40) except for item 9 (Supplementary Material, Table S3). Therefore, the four-factor structure with a correlated residual is the most appropriate model due to its parsimony (over the five-factor model) and interpretable factors.

Item 9 no longer meaningfully loaded onto any factor when including the correlated residual. The first factor (items 1–6) was interpreted as the repetitive motor behaviours factor and is the same factor identified by Barrett et al. (2015). The second factor (items 7, 8, 10–12) was interpreted as the interest in sensations and objects factor due to item content. The third factor (items 13–16) was interpreted as the insistence on sameness factor and is similar to the factor of the same name identified by Barrett et al. (2015). Lastly, the fourth factor (items 17–20) was interpreted as the restricted interests factor.

#### Confirmatory Factor Analyses

Overall, model fit indices suggested that the original Barrett et al. (2015) two-factor model provided poor model fit, whereas the four-factor model provided good model fit (Table 1). However, the four-factor model’s modification indices suggested that item 1 should cross-load on interest in sensations and objects. Removing this item continued to provide a good model fit for both the correlated model and the hierarchical variant (Table 1). The factors of the hierarchical model were well defined and had acceptable to excellent internal reliabilities (Table 2).^3^ The total score produced good to excellent internal reliability, *ω* = 0.94, *ω*_H_ = 0.80 (Supplementary Material Table S4).

**Table 1:** Fit Index Values of the RBQ-2A for the Tested Models in the University and Autistic Samples

Model/Sample	*χ*^*2*^ (df)	RMSEA [90% CI]	CFI	TLI
University sample				
Barrett et al. (2015)	291.308 (76)	.088 [.077, .099]	.945	.934
Orig. Four-factor model	323.555 (146)	.057 [.049, .066]	.963	.957
Mod. Four-factor model	227.212 (129)	.045 [.036, .055]	.977	.973
Hierarchical four-factor model	253.298 (131)	.050 [.041, .060]	.972	.967
Online autistic sample				
Barrett et al. (2015)	271.005 (76)	.095 [.083, .108]	.903	.884
Four-factor model	197.182 (129)	.043 [.031, .055]	.974	.969
Hierarchical four-factor model	231.335 (131)	.052 [.041, .063]	.961	.955

*χ*^*2*^ Chi-square, *df* degrees of freedom, *RMSEA* root mean square error of approximation, *90% CI* 90% confidence interval, *CFI* Comparative fit index, *TLI* Tucker Lewis index, *Orig* original four-factor model, retaining item 1, *Mod* Modification of the four-factor model where item 1 was removed

**Table 2:** Items, factor loadings, residual variance, and internal consistencies of the four-factor hierarchical model of the RBQ-2A in the university and online autistic samples

Item/Factor	University sample		Autistic sample	
	*λ*	*ε*	*λ*	*ε*
*Repetitive Motor Behaviours* (University Sample: *λ*_*RRB*_ = .68, *ε* = .54; *α* = .81, *ω* = .88; Autistic Sample: *λ*_*RRB*_ = .58, *ε* = .66; *α* = .76, *ω* = .82)				
1. Like to arrange items in rows or patterns?	Not included in analysis			
2. Repetitively fiddle with items? (e.g., spin, twiddle, bang, tap, twist, or flick anything repeatedly?)	.84	.70	.74	.45
3. Spin yourself around and around?	.61	.38	.63	.60
4. Rock backwards and forwards, or side to side, either when sitting or when standing?	.73	.53	.76	.43
5. Pace or move around repetitively (e.g., walk to and fro across a room, or around the same path in the garden?)	.75	.56	.64	.59
6. Make repetitive hand and/or finger movements? (e.g., flap, wave, or flick your hands or fingers repetitively?)	.88	.78	.73	.46
*Interest in Sensations & Objects* (University Sample: λ_RRB_ = .82, ε = .33; α = .76, ω = .85; Autistic Sample: λ_RRB_ = .82, ε = .33; α = .73, ω = .80)				
7. Have a fascination with specific objects (e.g., trains, road signs, or other things?)	.85	.71	.68	.54
8. Like to look at objects from particular or unusual angles?	.71	.51	.56	.68
9. Have a special interest in the smell of people or objects?	Not included in analysis			
10. Have a special interest in the feel of different surfaces?	.66	.44	.69	.52
11. Have any special objects you like to carry around?	.71	.50	.78	.40
12. Collect or hoard items of any sort?	.73	.53	.62	.62
*Insistence on Sameness* (University Sample: λ_RRB_ = .80, ε = .36; α = .86, ω = .90; Autistic Sample: λ_RRB_ = .79, ε = .38; α = .79, ω = .84)				
13. Insist on things at home remaining the same? (e.g., furniture staying in the same place, things being kept in certain places, or arranged in certain ways?)	.83	.69	.81	.34
14. Get upset about minor changes to objects (e.g., flecks of dirt on your clothes, minor scratches on objects?)	.80	.64	.73	.47
15. Insist that aspects of daily routine must remain the same?	.83	.68	.73	.47
16. Insist on doing things in a certain way or re-doing things until they’re “just right”?	.87	.75	.74	.45
*Restricted Interests* (University Sample: λ_RRB_ = .73, ε = .47; α = .72, ω = .80; Autistic Sample: λ_RRB_ = .88, ε = .23; α = .68, ω = .74)				
17. Play the same music, game or video, or read the same book repeatedly?	.81	.66	.69	.52
18. Insist on wearing the same clothes or refuse to wear new clothes?	.68	.47	.67	.55
19. Insist on eating the same foods, or a very small range of foods, at every meal?	.79	.63	.67	.55
20. What sort of activity will you choose if you are left to occupy yourself?	.54	.29	.54	.71

*λ* standardized factor loading, *λ*_*RRB*_ standardized factor loading onto general restricted and repetitive behavior factor, *ε* residual variance, *α* Cronbach’s alpha, *ω* McDonald’s omega

As a modification was made in the above model, an independent sample was collected to ensure the results could be replicated. This sample (*N* = 206; mean age of 20.20 years, SD = 4.54; 31.1% male, 67.5% female, 1.5% other) replicated the pattern of results shown above (Supplementary Material Tables S5 & S6).

Overall, the present results provide good evidence that the RBQ-2A reliably assesses four factors of RRBs and an overall RRB score. Items for the final four-factor model can be found in Table 2.

#### Convergent Validity

The descriptive statistics for the study measures are provided in the Supplementary Materials (Table S9). The four factors from the RBQ-2A showed numerically stronger relationships with the non-social scales than the social scales of other measures (Table 3), thus providing convergent and discriminant validity. Additionally, the RBQ-2A factors correlated moderately to highly with the sensory atypicality measure from the GSQ. Indeed, interest in sensations and objects accounted for 48% of the variance in sensory atypicality.

**Table 3:** Pearson Correlations between the RBQ-2A latent factors and other scales of social and non-social dimensions of autism in the university sample

	Social Dimensions		Non-Social Dimensions		
	Soc	Comm	Details	IU	Sens
Repetitive motor behaviours	.24**	.18*	.52**	.41**	.63**
Interest in sensations & objects	.34**	.18*	.68**	.43**	.69**
Insistence on sameness	.36**	.24**	.44**	.68**	.56**
Restrictive interests	.34**	.29**	.46**	.61**	.63**

*Soc* Social difficulties from the AQ, *Comm* Communication difficulties from the AQ, *IU* intolerance of uncertainty from the IUS, *Sens* Sensory Atypicality from the GSQ

^*^p < .01; **p < .001

## Study 2

### Methods

#### Participants and Procedure

Individuals on the spectrum were recruited through the online platform Prolific Academic which allows individuals to participate in online studies. Only individuals who reported to Prolific Academic as having a diagnosis of autism (although not independently verified) could view and enter our online study (accessed via Prolific Academic’s website). Individuals who reported self-identifying but not having a diagnosis could not enter the study. We confirmed that these individuals self-disclosed as being autistic by asking an additional screener question on whether they had a diagnosis (38 participants responded that they were either in the process of receiving a diagnosis, self-identified, did not have a diagnosis, or would rather not say, and so did not complete the questionnaire battery). Participants reporting being diagnosed as a child or as an adult provided demographic information and completed the online questionnaire battery.

Researchers commonly conceptualise autism on a continuum (e.g., Lundström et al., 2012; Ruzich et al., 2015), with autistic individuals falling on the high end of this distribution (Kamp-Becker et al., 2010; Ousley & Cermak, 2014). As there is substantial heterogeneity within the autistic population (Georgiades et al., 2013; Lord et al., 2020; Masi et al., 2017), participants scoring below the suggested cut-off on a measure of autistic traits (i.e., scores below 134 on the Comprehensive Autistic Trait Inventory; English et al., 2021), were not removed for the main analyses. While our sample showed clinically significant levels of autistic traits (*M* = 150.0, *SD* = 20.5), 20% were below this cut-off (*n* = 59), as such a sensitivity analysis which removes these individuals is included (see Supplementary Material Tables S10 & S11)). Overall, 283^4^ individuals on the spectrum composed the sample (mean age = 32.28 years, SD = 10.16, range: 18–62; male = 48.76%, female = 42.05%, gender-nonconforming = 9.19%; 41% American, 48% British, 13% European, with multiple ethnicities allowed). The sample consisted of 63.25% diagnosed as adults and 36.75% diagnosed as children. It is important to note that this sample was not matched with the sample from Study 1.

#### Materials

In addition to the RBQ-2A, participants completed the Comprehensive Autistic Trait Inventory (CATI; English et al., 2021). The 42-item CATI measures autistic traits across six subscales: (1) social interactions, (2) communication, (3) social camouflage, (4) repetitive behaviours, (5) cognitive rigidity, and (6) sensory sensitivity. Factor analyses have suggested that the first three subscales assess a social dimension of autism. The last three assess a non-social (i.e., RRBs) autism dimension (English et al., 2021). Participants respond to items on a five-point Likert scale: *Definitely disagree* (1) to *Definitely agree* (5). Higher scores indicate greater autistic traits. In the present study, the subscales showed good to excellent internal reliability (*α* ≥ 0.80).

#### Statistical Analysis

All statistical analyses were conducted in Mplus 8.0, except for obtaining Cronbach’s *α,* which was done via Jamovi 2.2.5. The same CFA analysis procedure from Study 1 was used, i.e., three CFAs were conducted: (1) the original factor structure from Barrett et al. (2015), (2) the four-factor model suggested from Study 1, and (3) a hierarchical variant of the four-factor model to assess whether the factors form an overall RRB score. Internal consistency reliabilities were obtained in the same fashion as Study 1.

Measurement invariance testing was conducted to determine whether the RBQ-2A showed potential psychometric biases between the two samples. This testing provides preliminary evidence on whether the RBQ-2A may be biased for individuals on the spectrum compared to those not with a diagnosis, as the two samples were not matched. Measurement invariance was tested with Mplus’ multiple group factor analysis convenience feature. Invariance was shown when the increasingly restrictive models did not show significantly worse model fit, i.e., ΔCFI or ΔTLI ≤ − 0.010 and ΔRMSEA ≥ 0.015 (Chen, 2007; Sass et al., 2014).

To assess convergent and discriminant validity, Pearson correlations were computed between the RBQ-2A latent factors and the subscales from the CATI. The latent factors of the RBQ-2A were obtained using the four-factor CFA, while the scales of the CATI were used as observed scores. It was expected that the RBQ-2A latent factors would show stronger relationships with the RRB subscales of the CATI (i.e., repetitive behaviours, cognitive rigidity, and sensory sensitivity) than with the social subscales (i.e., social interactions, communication, and social camouflage). Additionally, it was expected that the subscales and factors measuring similar constructs would have the strongest relationships (e.g., the repetitive behaviours scale from the CATI and the repetitive motor behaviours factor from the RBQ-2A).

### Results

#### Confirmatory Factor Analyses

The series of CFAs produced the same pattern of results as in Study 1, with the original Barrett et al. (2015) two-factor model providing poor model fit values, while the four-factor model and the hierarchical variant provided good model fit values (Table 1). The factors of the hierarchical model were well defined and produced acceptable to excellent internal reliabilities (Table 2). The total score produced good to excellent internal reliability, *ω* = 0.91, *ω*_H_ = 0.77.

Measurement invariance was tested between this online autistic sample and the unmatched university sample from Study 1 (Table 4; CFA results from the separate samples are found in Table 1). This testing provided evidence of configural, metric, and scalar invariance, suggesting that the RBQ-2A loaded onto the same latent factors and had equivalent factor loadings and intercepts across the study groups. However, as the two study groups were not matched on demographic variables, caution should be taken to generalize invariance across autistic and non-autistic groups. Nonetheless, the means of the factors were greater in the online autistic sample. The non-autistic sample was constrained to have means of 0, while the autistic sample had means significantly greater than 0 (i.e., mean of Repetitive Motor Behaviours = 0.9, *p* < 0.001; mean of Interest in sensation and object = 2.1, *p* < 0.001; mean of Insistence on sameness = 1.5, *p* < 0.001; mean of Restricted Interests = 1.2, *p* < 0.001).

**Table 4:** Measurement invariance between the university and online autistic samples

	*χ*^*2*^ (df)	RMSEA	CFI	TLI	ΔRMSEA	ΔCFI	ΔTLI
Configural	422.602 (258)	.044	.977	.973	–	–	–
Metric	461.424 (272)	.046	.973	.970	.002	− .004	− .003
Scalar	507.017 (298)	.046	.971	.970	.000	− .002	.000

*χ*^*2 *^Chi-square, *df* degrees of freedom, *RMSEA* root mean square error of approximation, *90% CI* 90% confidence interval, *CFI* Comparative fit index, *TLI* Tucker Lewis index, *Δ* difference compared to previous model

#### Convergent Validity

The descriptive statistics of the variables from the mimic model are available in the Supplementary Material (Table 9). The correlations between the RBQ-2A latent variables and the CATI subscales produced the expected pattern of results (Table 5).^5^ Overall, the RBQ-2A factors showed numerically stronger relationships with the RRB subscales compared to the social subscales of the CATI. Additionally, the repetitive behaviours subscale from the CATI had its largest correlation with the repetitive motor behaviours factor from the RBQ-2A. Also, the cognitive rigidity CATI subscale had its largest correlation with the insistence on sameness factor (and, to a lesser extent, restricted interests) from the RBQ-2A. Lastly, the sensory sensitivity CATI subscale had its largest correlation with the interest in sensations and objects factor from the RBQ-2A.

**Table 5:** RBQ-2A factors correlations with CATI subscales in the online autistic sample

	CATI Subscales					
Factors	REP (*n* = 281)	RIG (*n* = 280)	SEN (*n* = 282)	SOC (*n* = 282)	COM (*n* = 280)	CAM (*n* = 282)
RMB	.81***	.29***	.40***	.17*	.09	.39***
ISO	.62***	.53***	.57***	.30***	.30***	.41***
IS	.40***	.79***	.45***	.42***	.45***	.36***
RI	.50***	.63***	.49***	.48***	.54***	.42***

*CATI* Comprehensive Autistic Trait Inventory, *Rep* Repetitive Behaviours, *Rig* Cognitive Rigidity, *SEN* Sensory Sensitivity, *SOC* Social Interactions, *COM* Communication, *CAM* Social Camouflage. *RMB* Repetitive Motor Behaviours from RBQ-2A, *ISO* Interest in Sensations and Objects from RBQ-2A, *IS* Insistence on Sameness from RBQ-2A, *RI* Restricted Interests from RBQ-2A

^*^p < .05; **p < .01; ***p < .001

## Discussion

The present study explored the factor structure of the RBQ-2A and how these factors relate to other autistic traits. Factor analyses revealed that, within both an online autistic and non-autistic sample, the RBQ-2A contained four factors: (1) repetitive motor behaviours—items 2–6, (2) interest in sensations and objects—items 7, 8, 10–12, (3) insistence on sameness—items 13–16, and (4) restricted interests—items 17–20. Items 1 and 9 should not be included in calculating the subscales or total scores but the full RBQ-2A should be administered as these items may still be beneficial clinically. In addition, the RBQ-2A showed evidence of measurement invariance between autistic and non-autistic individuals. The factor structure is consistent with past studies (e.g., Barrett et al., 2015, 2018) in eliciting the original factors (i.e., repetitive motor behaviours and insistence on sameness) but also extends the structure by suggesting two additional factors (i.e., interest in sensations and objects, and restricted interests). Furthermore, in line with conceptualisations suggesting the presence of broader social and non-social dimensions of autism (e.g., English et al., 2021; Shuster et al., 2014; Warrier et al., 2019), a higher-order RRB construct was found. Thus, while RRBs contain four distinct factors, they can be represented under one construct.

The DSM-5 and other research (e.g., Grove et al., 2021) suggest that RRBs contain (1) repetitive motor movements, (2) insistence on sameness, (3) restricted interests, and (4) sensory sensitivity/interest in sensory aspects of the environment. The present results suggest that the RBQ-2A shows construct validity to assess RRBs across these four criteria/factors in both a university and an autistic population. Furthermore, these factors followed the relationships expected with other measures of social and non-social autistic dimensions, providing evidence of convergent validity (e.g., strong relationships with other RRB measures) and discriminant validity compared to non-social autistic traits (e.g., social and communication difficulties).

Overall, this new four-factor structure of the RBQ-2A allows for greater differentiation of the RRBs, which will likely benefit future research interested in how RRBs relate to other constructs such as anxiety (e.g., Sellick et al., 2021) and social cognition (e.g., Brett & Maybery, 2022). Furthermore, the scale structure provides additional opportunities within clinical settings. By finding that the RBQ-2A is capable of measuring RRBs overall, it can be used by clinicians wanting to assess the levels of particular behaviours being exhibited by an individual or screen potential individuals for autism, and could potentially aid in clinical formulations. Additionally, given that different RRBs have mixed relationships with intolerance of uncertainty and anxiety (Joyce et al., 2017), the current paper suggests that the RBQ-2A may be used to assess which specific behaviours (e.g., insistence on sameness rather than repetitive motor behaviours) may predict greater/reduced intolerance of uncertainty and anxiety, thus aiding intervention.

While these repetitive and restricted behaviours represent a variety of different behaviours (e.g., repetitive motor behaviours and insistence on sameness), the current finding of a hierarchical structure provides evidence that these different behaviours have commonality among both autistic and non-autistic samples. This provides support for a unified dimension of autism encapsulating non-social behaviours (i.e., repetitive motor behaviours, interest in sensations and objects, insistence on sameness, & restricted interests). Future research may explore whether additional non-social behaviours linked with autism (e.g., intolerance of uncertainty) are a component of this non-social autistic dimension or are just closely related.

This manuscript will benefit researchers and clinicians attempting to measure RRBs, although there are some limitations. Given the online autistic sample, there should be caution in generalising the current findings to autistic individuals less likely to be recruited online, such as individuals with an intellectual disability (see Rødgaard et al., 2022). As such, the findings here should not be generalised to other autistic populations (e.g., “profound autism”; Lord et al., 2022). Nonetheless, the findings suggest that other online studies recruiting autistic individuals will benefit from using this factor structure of the RBQ-2A. Another limitation is that the two studies used unmatched samples. As such, direct comparisons of autistic and non-autistic groups should be approached with caution as differences in demographic variables (e.g., gender, age, and ethnicity) were not controlled for in the testing of measurement invariance. Future research should conduct additional testing of measurement invariance with matched samples to provide greater confidence in the RBQ-2A’s invariance.

The RBQ-2A factor structure identified here is consistent with the DSM-5 and reports from other research (e.g., Grove et al., 2021) by providing evidence of four factors: repetitive motor behaviours, interest in sensation and objects, insistence on sameness, and restricted interests. Furthermore, to our knowledge, this is the first study that assessed and found that these factors form a general RRB construct. Thus, the results help advance the assessment of RRBs by validating the RBQ-2A to assess total RRBs across a wide variety of behaviours within autistic and non-autistic populations.

### Supplementary Information

Below is the link to the electronic supplementary material.

Supplementary file1 (XLSX 279 kb)

Supplementary file2 (DOCX 61 kb)

## Footnotes

1 Initially there were 389 participants, although 21 participants were removed because they self-reported invalid data (i.e., responding “No, my data is NOT valid and should not be used for research” after completing the questionnaires; *n* = 12) or completed the questionnaires quickly, likely reflecting inattentive responding (< 2 s per item, *n* = 9).

2 Participants could choose multiple ethnicities.

3 An alternative scoring method previously reported for the RBQ-2A collapses the Likert-scales so that all items are on a three-point scale (Barrett et al., 2015, 2018). When using this scoring method, the pattern of results was replicated (Supplementary Material, Tables S7 & S8).

4 Originally there were 286 participants, although three participants reported having invalid data.

5 Some participants had missing data and were removed; the sample size for each correlation is found in Table 4
