# Supplementary material for: Assessing Restricted and Repetitive Behaviours in Online-Sampled Autistic and Non-autistic Individuals: Factor Structure of the Repetitive Behaviours Questionnaire for Adults (RBQ-2A)
Source: J Autism Dev Disord. 2023 Apr 13;54(6):2138–47. doi: 10.1007/s10803-023-05977-w (PMC11142953; doi:10.1007/s10803-023-05977-w)
Supplement: Supplementary file 2 — Supplementary file2 (DOCX 61 kb) [file 10803_2023_5977_MOESM2_ESM.docx]

**Supplementary Material**

**Series of ESEMs**

Table S1. Model fit indices from the ESEMs for Study 1

| No. of Factors | Chi-square (df) | RMSEA [90% CI] | CFI | TLI | ΔRMSEA | ΔCFI | ΔTLI |
| --- | --- | --- | --- | --- | --- | --- | --- |
| 1 Factor | 984.232* (170) | .114* [.107/.121] | .833 | .814 | - | - | - |
| 2 Factor | 562.624* (151) | .086* [.079/.094] | .916 | .894 | -.028 | .083 | .080 |
| 3 Factor | 336.837* (133) | .065* [.056/.073] | .958 | .940 | -.021 | .042 | .046 |
| 4 Factor | 202.586* (116) | .045 [.035/.055] | .982 | .971 | -.020 | .024 | .031 |
| 5 Factor | 129.235* (100) | .028 [.010/.041] | .994 | .989 | -.017 | .012 | .018 |
| 4 Factor W/ correlated residuals | 169.252* (115) | .036 [.024/.047] | .989 | .982 | .008 | .005 | .007 |

*Note*: RMSEA = root mean square error of approximation; CFI = comparative fit index; TLI = Tucker Lewis index; Δ = change compared to previous model.

*Indicates *p*< .05.

Table S2. Inter-factor correlations from the ESEMs for Study 1

| 2 Factors:   \|  \| F2 \| \| --- \| --- \| \| F1 \| .48* \| | 3 Factors:   \|  \| F2 \| F3 \| \| --- \| --- \| --- \| \| F1 \| .35* \| .25* \| \| F2 \| - \| .19* \| | 4 Factors:   \|  \| F2 \| F3 \| F4 \| \| --- \| --- \| --- \| --- \| \| F1 \| .50* \| .37* \| .36* \| \| F2 \| - \| .49* \| .30* \| \| F3 \|  \| - \| .48* \| | 5 Factors:   \|  \| F2 \| F3 \| F4 \| F5 \| \| --- \| --- \| --- \| --- \| --- \| \| F1 \| .49* \| .36* \| .40* \| .42* \| \| F2 \| - \| .46* \| .31* \| .36* \| \| F3 \|  \| - \| .51* \| .32* \| \| F4 \|  \|  \| - \| .22* \| | 4 Factors W/cr:   \|  \| F2 \| F3 \| F4 \| \| --- \| --- \| --- \| --- \| \| F1 \| .51* \| .39* \| .36* \| \| F2 \| - \| .49* \| .29* \| \| F3 \|  \| - \| .47* \| |
| --- | --- | --- | --- | --- | --- | --- | --- | --- | --- | --- | --- | --- | --- | --- | --- | --- | --- | --- | --- | --- | --- | --- | --- | --- | --- | --- | --- | --- | --- | --- | --- | --- | --- | --- | --- | --- | --- | --- | --- | --- | --- | --- | --- | --- | --- | --- | --- | --- | --- | --- | --- | --- | --- | --- | --- | --- | --- | --- | --- | --- | --- | --- | --- | --- | --- | --- | --- | --- | --- | --- | --- | --- | --- | --- |

*Note*: W/cr = with correlated residuals; F1 = Factor 1; F2 = Factor 2; F3 = Factor 3; F4 = Factor 4; F5 = Factor 5.

*Indicates *p*< .05.

Table S3. Factor Loadings for Study 1

| 1 Factor   \| Item \| F1 \| \| --- \| --- \| \| 1 \| **.69** \| \| 2 \| **.72** \| \| 3 \| **.49** \| \| 4 \| **.62** \| \| 5 \| **.63** \| \| 6 \| **.75** \| \| 7 \| **.72** \| \| 8 \| **.62** \| \| 9 \| **.51** \| \| 10 \| **.59** \| \| 11 \| **.59** \| \| 12 \| **.61** \| \| 13 \| **.75** \| \| 14 \| **.73** \| \| 15 \| **.73** \| \| 16 \| **.78** \| \| 17 \| **.60** \| \| 18 \| **.51** \| \| 19 \| **.59** \| \| 20 \| **.40** \| | A | 2 Factors   \| Item \| F1 \| F2 \| \| --- \| --- \| --- \| \| 1 \| **.53** \| .28 \| \| 2 \| **.81** \| .00 \| \| 3 \| **.78** \| -.21 \| \| 4 \| **.76** \| -.07 \| \| 5 \| **.73** \| -.01 \| \| 6 \| **.79** \| .07 \| \| 7 \| **.46** \| **.40** \| \| 8 \| **.51** \| .22 \| \| 9 \| **.41** \| .20 \| \| 10 \| **.52** \| .17 \| \| 11 \| .30 \| **.40** \| \| 12 \| .36 \| .36 \| \| 13 \| .00 \| **.82** \| \| 14 \| .05 \| **.76** \| \| 15 \| -.11 \| **.89** \| \| 16 \| .16 \| **.73** \| \| 17 \| .16 \| **.54** \| \| 18 \| .03 \| **.55** \| \| 19 \| -.03 \| **.69** \| \| 20 \| -.03 \| **.48** \| | A | 3 Factors   \| Item \| F1 \| F2 \| F3 \| \| --- \| --- \| --- \| --- \| \| 1 \| **.44** \| **.46** \| .01 \| \| 2 \| **.80** \| .07 \| -.01 \| \| 3 \| **.74** \| .00 \| -.20 \| \| 4 \| **.78** \| -.04 \| -.02 \| \| 5 \| **.75** \| -.02 \| .03 \| \| 6 \| **.77** \| .13 \| .03 \| \| 7 \| .27 \| **.70** \| -.02 \| \| 8 \| .36 \| **.52** \| -.08 \| \| 9 \| .35 \| .30 \| .04 \| \| 10 \| **.40** \| **.43** \| -.07 \| \| 11 \| .19 \| **.52** \| .11 \| \| 12 \| .24 \| **.52** \| .07 \| \| 13 \| .01 \| **.55** \| **.51** \| \| 14 \| -.01 \| **.63** \| **.41** \| \| 15 \| -.05 \| **.49** \| **.64** \| \| 16 \| .19 \| **.45** \| **.49** \| \| 17 \| .34 \| .02 \| **.54** \| \| 18 \| .32 \| -.19 \| **.63** \| \| 19 \| .20 \| .01 \| **.68** \| \| 20 \| .15 \| -.04 \| **.51** \| | A | 4 Factors   \| Item \| F1 \| F2 \| F3 \| F4 \| \| --- \| --- \| --- \| --- \| --- \| \| 1 \| **.40** \| .34 \| .26 \| -.13 \| \| 2 \| **.78** \| .06 \| -.01 \| .04 \| \| 3 \| **.70** \| .17 \| -.28 \| .01 \| \| 4 \| **.83** \| -.11 \| .00 \| .01 \| \| 5 \| **.77** \| -.09 \| .02 \| .07 \| \| 6 \| **.79** \| .02 \| .12 \| .00 \| \| 7 \| .02 \| **.82** \| .06 \| .04 \| \| 8 \| .19 \| **.64** \| -.02 \| -.01 \| \| 9 \| .23 \| .35 \| .02 \| .07 \| \| 10 \| .29 \| **.49** \| .01 \| -.03 \| \| 11 \| -.09 \| **.70** \| -.02 \| .21 \| \| 12 \| .05 \| **.60** \| .06 \| .10 \| \| 13 \| .03 \| .07 \| **.77** \| .04 \| \| 14 \| .00 \| .20 \| **.73** \| -.04 \| \| 15 \| -.03 \| -.05 \| **.82** \| .16 \| \| 16 \| .20 \| .03 \| **.65** \| .13 \| \| 17 \| .15 \| .07 \| .06 \| **.61** \| \| 18 \| .09 \| -.11 \| -.11 \| **.80** \| \| 19 \| -.07 \| .11 \| .09 \| **.74** \| \| 20 \| .03 \| -.08 \| .15 \| **.49** \| | A | 5 Factors   \| Item \| F1 \| F2 \| F3 \| F4 \| F5 \| \| --- \| --- \| --- \| --- \| --- \| --- \| \| 1 \| **.43** \| .34 \| .27 \| -.14 \| .00 \| \| 2 \| **.82** \| .10 \| -.03 \| .04 \| -.07 \| \| 3 \| **.63** \| .07 \| -.27 \| .01 \| .23 \| \| 4 \| **.79** \| -.19 \| .01 \| .00 \| .15 \| \| 5 \| **.73** \| -.16 \| .02 \| .06 \| .15 \| \| 6 \| **.80** \| .04 \| .11 \| -.01 \| -.02 \| \| 7 \| .03 \| **.85** \| .05 \| .05 \| .02 \| \| 8 \| .08 \| **.49** \| -.01 \| .00 \| .37 \| \| 9 \| -.01 \| .02 \| .04 \| .11 \| **.74** \| \| 10 \| .15 \| .28 \| .03 \| -.03 \| **.49** \| \| 11 \| -.07 \| **.71** \| -.03 \| .23 \| .01 \| \| 12 \| .06 \| **.59** \| .07 \| .11 \| .03 \| \| 13 \| .03 \| .02 \| **.77** \| .04 \| .08 \| \| 14 \| .00 \| .16 \| **.74** \| -.05 \| .07 \| \| 15 \| -.02 \| -.07 \| **.82** \| .16 \| .02 \| \| 16 \| .24 \| .06 \| **.65** \| .12 \| -.09 \| \| 17 \| .06 \| -.02 \| .05 \| **.64** \| .19 \| \| 18 \| .06 \| .00 \| -.14 \| **.82** \| -.04 \| \| 19 \| -.13 \| .07 \| .07 \| **.77** \| .09 \| \| 20 \| .07 \| .00 \| .12 \| **.50** \| -.19 \| | A | 4 Factors W/ correlated residual   \| Item \| F1 \| F2 \| F3 \| F4 \| \| --- \| --- \| --- \| --- \| --- \| \| 1 \| **.41** \| .34 \| .24 \| -.13 \| \| 2 \| **.79** \| .05 \| -.03 \| .04 \| \| 3 \| **.72** \| .15 \| -.29 \| .01 \| \| 4 \| **.84** \| -.14 \| .00 \| .01 \| \| 5 \| **.78** \| -.10 \| .01 \| .06 \| \| 6 \| **.79** \| .02 \| .10 \| .00 \| \| 7 \| .03 \| **.84** \| .03 \| .05 \| \| 8 \| .21 \| **.62** \| -.02 \| -.01 \| \| 9 \| .25 \| .22 \| .08 \| .09 \| \| 10 \| .31 \| **.42** \| .05 \| -.04 \| \| 11 \| -.08 \| **.71** \| -.03 \| .22 \| \| 12 \| .06 \| **.61** \| .05 \| .11 \| \| 13 \| .04 \| .07 \| **.77** \| .04 \| \| 14 \| .00 \| .20 \| **.73** \| -.05 \| \| 15 \| -.03 \| -.05 \| **.83** \| .16 \| \| 16 \| .20 \| .04 \| **.64** \| .12 \| \| 17 \| .16 \| .05 \| .08 \| **.61** \| \| 18 \| .09 \| -.02 \| -.10 \| **.79** \| \| 19 \| -.06 \| .10 \| .11 \| **.74** \| \| 20 \| .03 \| -.07 \| .15 \| **.48** \| |
| --- | --- | --- | --- | --- | --- | --- | --- | --- | --- | --- | --- | --- | --- | --- | --- | --- | --- | --- | --- | --- | --- | --- | --- | --- | --- | --- | --- | --- | --- | --- | --- | --- | --- | --- | --- | --- | --- | --- | --- | --- | --- | --- | --- | --- | --- | --- | --- | --- | --- | --- | --- | --- | --- | --- | --- | --- | --- | --- | --- | --- | --- | --- | --- | --- | --- | --- | --- | --- | --- | --- | --- | --- | --- | --- | --- | --- | --- | --- | --- | --- | --- | --- | --- | --- | --- | --- | --- | --- | --- | --- | --- | --- | --- | --- | --- | --- | --- | --- | --- | --- | --- | --- | --- | --- | --- | --- | --- | --- | --- | --- | --- | --- | --- | --- | --- | --- | --- | --- | --- | --- | --- | --- | --- | --- | --- | --- | --- | --- | --- | --- | --- | --- | --- | --- | --- | --- | --- | --- | --- | --- | --- | --- | --- | --- | --- | --- | --- | --- | --- | --- | --- | --- | --- | --- | --- | --- | --- | --- | --- | --- | --- | --- | --- | --- | --- | --- | --- | --- | --- | --- | --- | --- | --- | --- | --- | --- | --- | --- | --- | --- | --- | --- | --- | --- | --- | --- | --- | --- | --- | --- | --- | --- | --- | --- | --- | --- | --- | --- | --- | --- | --- | --- | --- | --- | --- | --- | --- | --- | --- | --- | --- | --- | --- | --- | --- | --- | --- | --- | --- | --- | --- | --- | --- | --- | --- | --- | --- | --- | --- | --- | --- | --- | --- | --- | --- | --- | --- | --- | --- | --- | --- | --- | --- | --- | --- | --- | --- | --- | --- | --- | --- | --- | --- | --- | --- | --- | --- | --- | --- | --- | --- | --- | --- | --- | --- | --- | --- | --- | --- | --- | --- | --- | --- | --- | --- | --- | --- | --- | --- | --- | --- | --- | --- | --- | --- | --- | --- | --- | --- | --- | --- | --- | --- | --- | --- | --- | --- | --- | --- | --- | --- | --- | --- | --- | --- | --- | --- | --- | --- | --- | --- | --- | --- | --- | --- | --- | --- | --- | --- | --- | --- | --- | --- | --- | --- | --- | --- | --- | --- | --- | --- | --- | --- | --- | --- | --- | --- | --- | --- | --- | --- | --- | --- | --- | --- | --- | --- | --- | --- | --- | --- | --- | --- | --- | --- | --- | --- | --- | --- | --- | --- | --- | --- | --- | --- | --- | --- | --- | --- | --- | --- | --- | --- | --- | --- | --- | --- | --- | --- | --- | --- | --- | --- | --- | --- | --- | --- | --- | --- | --- | --- | --- | --- | --- | --- | --- | --- | --- | --- | --- | --- | --- | --- | --- | --- | --- | --- | --- | --- | --- | --- | --- | --- | --- | --- | --- | --- | --- | --- | --- | --- | --- | --- | --- | --- | --- | --- | --- | --- | --- | --- | --- | --- | --- | --- | --- | --- | --- | --- | --- | --- | --- | --- | --- | --- | --- | --- | --- | --- | --- | --- | --- | --- | --- | --- | --- | --- | --- | --- | --- | --- | --- | --- | --- | --- | --- | --- | --- | --- | --- | --- | --- | --- | --- | --- | --- | --- | --- | --- | --- | --- | --- | --- | --- | --- | --- | --- | --- | --- | --- | --- | --- | --- | --- | --- | --- | --- | --- | --- | --- | --- | --- | --- | --- | --- | --- | --- | --- | --- | --- | --- | --- | --- | --- | --- | --- | --- | --- | --- | --- | --- | --- | --- | --- | --- | --- | --- | --- | --- | --- | --- | --- | --- | --- | --- |

*Note*: Bold indicates factor loadings > |.40|. F = Factor No.

**Confirmatory Factor Analyses:**

***Bi-factor model***

This model was used to obtain omega and hierarchical omega of the total RRB score in both **Study 1** and **Study 2**. It showed good model fit across all indices (Study 1: RMSEA = .049, CFI = .976, TLI = .968; Study 2: RMSEA = .050, CFI = .968, TLI = .959). The factor loadings and residual variance for the two data sets are shown in Table S4.

Table S4. Bi-factor Factor Loadings from Studies 1 & 2

|  | Study 1 | | | *A* | Study 2 | | |
| --- | --- | --- | --- | --- | --- | --- | --- |
| Item/*Factor* | *λ_S_* | *λ_G_* | *ε* |  | *λ_S_* | *λ_G_* | *ε* |
| *Repetitive Motor Behaviours* | | | | | | | |
| 2 | .58 | .59 | .32 |  | .53 | .47 | .50 |
| 3 | .59 | .35 | .53 |  | .70 | .27 | .57 |
| 4 | .62 | .46 | .41 |  | .57 | .46 | .54 |
| 5 | .58 | .49 | .43 |  | .54 | .36 | .42 |
| 6 | .56 | .64 | .28 |  | .59 | .43 | .54 |
| *Interest in sensations & objects* | | | | | | | |
| 7 | .58 | .67 | .21 |  | .45 | .54 | .49 |
| 8 | .42 | .58 | .49 |  | .60 | .40 | .52 |
| 10 | .24 | .57 | .62 |  | .40 | .56 | .48 |
| 11 | .47 | .57 | .46 |  | .21 | .69 | .52 |
| 12 | .38 | .60 | .50 |  | .35 | .50 | .38 |
| *Insistence on Sameness* | | | | | | | |
| 13 | .58 | .63 | .27 |  | .53 | .63 | .68 |
| 14 | .49 | .64 | .36 |  | .31 | .62 | .48 |
| 15 | .60 | .62 | .25 |  | .50 | .55 | .55 |
| 16 | .36 | .75 | .30 |  | .53 | .56 | .59 |
| *Restricted Interests* | | | | | | | |
| 17 | .42 | .62 | .45 |  | .10 | .64 | .42 |
| 18 | .66 | .44 | .36 |  | .55 | .57 | .63 |
| 19 | .54 | .57 | .38 |  | .39 | .58 | .49 |
| 20 | .37 | .40 | .71 |  | .14 | .50 | .27 |

*Note:* *λ_S_* = Specific factor loading, *λ_G_* = General factor loading, *ε* = residual variance.

**Replication Study**

In the replication study, 206 participants^[[1]](#footnote-1)^ with a mean age of 20.20 years (SD = 4.54; 31.1% male, 67.5% female, 1.5% gender-nonconforming), completed a series of questionnaires including the RBQ-2A through the online platform Qualtrics.

This sample produced a similar pattern of results to what was found in Study 1. The original Barrett et al. (2015) two-factor structure did not show good model fit, and interestingly the original four-factor structure also did not show good model fit. After removing item 1 (as suggested in Study 1), the four-factor structure and the hierarchical variant produced good model fit indices (Table S5). Additionally, these factors were well defined (Table S6).

Table S5. Model fit indices from the Confirmatory Factor Analyses in the replication study

| Models | Chi-square (df) | RMSEA [90% CI] | CFI | TLI |
| --- | --- | --- | --- | --- |
| Barrett et al., 2015 | 186.341* (76) | .084* [.069/.099] | .926 | .912 |
| Four-factor | 251.946* (146) | .059 [.047/.072] | .945 | .935 |
| Removing item 1 | 193.007* (129) | .049 [.034, .063] | .964 | .957 |
| Hierarchical four-factor | 196.112* (131) | .049 [.034, .063] | .963 | .957 |

*Note*: RMSEA = root mean square error of approximation; CFI = comparative fit index; TLI = Tucker Lewis index; Δ = change compared to previous model.

*Indicates *p*< .05.

Table S6. Factor Loadings from the Four-factor Model in the replication study

| *Factor*/Item No. | *λ* | *ε* |
| --- | --- | --- |
| *Repetitive Motor Behaviours* | .67^a^ | .55^a^ |
| 2 | .72 | .49 |
| 3 | .70 | .51 |
| 4 | .72 | .48 |
| 5 | .81 | .34 |
| 6 | .84 | .30 |
| *Interest in Sensations & Objects* | .76^a^ | .42^a^ |
| 7 | .79 | .37 |
| 8 | .69 | .52 |
| 10 | .60 | .64 |
| 11 | .55 | .70 |
| 12 | .56 | .69 |
| *Insistence on Sameness* | .77^a^ | .41^a^ |
| 13 | .67 | .55 |
| 14 | .64 | .59 |
| 15 | .70 | .51 |
| 16 | .88 | .22 |
| *Restricted Interests* | .88^a^ | .22^a^ |
| 17 | .75 | .44 |
| 18 | .82 | .34 |
| 19 | .69 | .53 |
| 20 | .38 | .85 |

*Note*: *λ* = Factor loading, *ε* = residual variance. ^a^Indicates second-order factor loading, or residual variance.

**Collapsing the Likert-scales**

As some scoring methods suggest collapsing the Likert-scales to all be 3-point scales (e.g., Barrett et al., 2015, 2018), an additional analysis was completed with this scoring procedure. The Study 1 sample was used for this analysis. The pattern of results are consistent with those reported for Study 1.

Table S7. Model fit from the Confirmatory Factor Analyses for the re-analysis of Study 1 data using 3-point scales

| Models | Chi-square (df) | RMSEA [90% CI] | CFI | TLI |
| --- | --- | --- | --- | --- |
| Barrett et al., 2015 | 258.325* (325) | .081* [.070, .092] | .943 | .932 |
| Four-factor | 287.477* (146) | .051 [.043, .060] | .966 | .960 |
| Removing item 1 | 208.191* (129) | .041 [.030, .051] | .979 | .975 |
| Hierarchical four-factor | 229.837* (131) | .045 [.035, .055] | .974 | .969 |

*Note*: RMSEA = root mean square error of approximation; CFI = comparative fit index; TLI = Tucker Lewis index; Δ = change compared to previous model.

*Indicates *p*< .05.

Table S8. Factor Loadings from the Four-factor Model for the re-analysis of Study 1 data using 3-point scales

| *Factor*/Item No. | *λ* | *ε* |
| --- | --- | --- |
| *Repetitive Motor Behaviours* | .69^a^ | .53^a^ |
| 2 | .83 | .31 |
| 3 | .62 | .62 |
| 4 | .72 | .49 |
| 5 | .73 | .47 |
| 6 | .88 | .23 |
| *Interest in Sensations & Objects* | .82^a^ | .32^a^ |
| 7 | .84 | .29 |
| 8 | .72 | .48 |
| 10 | .66 | .57 |
| 11 | .71 | .50 |
| 12 | .72 | .48 |
| *Insistence on Sameness* | .80^a^ | .37^a^ |
| 13 | .82 | .33 |
| 14 | .80 | .35 |
| 15 | .81 | .34 |
| 16 | .84 | .30 |
| *Restricted Interests* | .71^a^ | .50^a^ |
| 17 | .81 | .34 |
| 18 | .69 | .53 |
| 19 | .81 | .34 |
| 20 | .55 | .70 |

*Note*: *λ* = Factor loading, *ε* = residual variance. ^a^Indicates second-order factor loading, or residual variance.

Table S9. Descriptive Statistics for the study variables in the mimic models from Studies 1 and 2.

| Variables/*Measure* | *M* | *SD* |
| --- | --- | --- |
| **Study 1** | | |
| *RBQ-2A* |  |  |
| Repetitive motor behaviours | 0 | 0.7 |
| Interest in sensations & objects | 0 | 0.7 |
| Insistence on sameness | 0 | 0.7 |
| Restrictive Interests | 0 | 0.6 |
| *AQ* |  |  |
| Social difficulties | 29.0 | 7.3 |
| Communication difficulties | 15.4 | 3.3 |
| Attention to details | 16.6 | 4.4 |
| *GSQ* |  |  |
| Atypical sensory responsiveness | 88.7 | 21.4 |
| *IUS-12* |  |  |
| Intolerance of uncertainty | 30.8 | 10.8 |
| **Study 2** | | |
| *RBQ-2A* |  |  |
| Repetitive motor behaviours | 0 | 0.5 |
| Interest in sensations & objects | 0 | 0.4 |
| Insistence on sameness | 0 | 0.6 |
| Restrictive Interests | 0 | 0.5 |
| *CATI* |  |  |
| Repetitive behaviours | 26.3 | 5.7 |
| Cognitive Rigidity | 28.0 | 4.9 |
| Sensory Sensitivity | 26.2 | 6.4 |
| Social Interactions | 27.6 | 6.4 |
| Communication | 22.8 | 5.8 |
| Social Camouflage | 25.6 | 6.0 |

*Note*: RBQ-2A = Repetitive Behaviours Questionnaire for adults; AQ = Autism-Spectrum Quotient; GSQ = Glasgow Sensory Questionnaire; IUS-12 = Intolerance of Uncertainty Scale—short form; CATI = Comprehensive Autistic Trait Inventory.

Sensitivity Analyses:

Removing autistic individuals based on scoring below specified cut-off of the CATI (i.e., below 134).

59 participants were removed from the online autistic sample for scoring below the cut-off, leaving 224 participants. Re-running the analyses with this sample produced the same pattern of results within the main manuscript (see Tables S10 & S11). The total score showed good internal consistency reliability (*ω* = .87; *ω_H_* = .66).

Table S10. Fit Index Values of the RBQ-2A for the Tested Models in the online autistic sample (removing individuals below CATI cut-off).

| Models | *χ^2^* (df) | RMSEA [90% CI] | CFI | TLI |
| --- | --- | --- | --- | --- |
| Barrett et al. (2015) | 235.957 (76) | .097 [.083, .111] | .854 | .825 |
| Four-factor model | 194.702 (129) | .048 [.033, .061] | .953 | .944 |
| Hierarchical four-factor model | 227.329 (131) | .057 [.045, .070] | .931 | .919 |
| Bi-factor model | 195.613 (117) | .055 [.041, .068] | .944 | .926 |

Table S11. Items, factor loadings, residual variance, and internal consistencies of the four-factor hierarchical model and bi-factor of the RBQ-2A in the online autistic sample (removing individuals below CATI cut-off).

|  | *Four-factor* | |  | *Bi-factor* | | |
| --- | --- | --- | --- | --- | --- | --- |
| Item/*Factor* | *λ* | *ε* |  | *λ_S_* | *λ_G_* | *ε* |
| *Repetitive Motor Behaviours* (*λ_RRB_* = .48; *ε* = .77; *ω* = .80) | | | | | | |
|  | Not Included | | | | | |
|  | .69 | .53 |  | .54 | .38 | .57 |
|  | .63 | .60 |  | .73 | .18 | .43 |
|  | .75 | .44 |  | .58 | .42 | .49 |
|  | .62 | .62 |  | .55 | .29 | .61 |
|  | .67 | .55 |  | .61 | .29 | .53 |
| *Interest in Sensations and Objects* (*λ_RRB_* = .78; *ε* = .40; *ω* = .79) | | | | | | |
|  | .65 | .58 |  | .56 | .45 | .48 |
|  | .55 | .70 |  | .58 | .34 | .55 |
|  | Not Included | | | | | |
|  | .65 | .58 |  | .42 | .50 | .58 |
|  | .81 | .34 |  | .51 | .73 | .42 |
|  | .63 | .60 |  | .51 | .44 | .54 |
| *Insistence on Sameness* (*λ_RRB_* = .70; *ε* = .51; *ω* = .80) | | | | | | |
|  | .76 | .43 |  | .60 | .52 | .38 |
|  | .73 | .47 |  | .40 | .56 | .53 |
|  | .65 | .58 |  | .48 | .44 | .57 |
|  | .71 | .50 |  | .55 | .47 | .48 |
| *Restricted Interests* (*λ_RRB_* = .77; *ε* = .40; *ω* = .69) | | | | | | |
|  | .69 | .53 |  | .19 | .58 | .63 |
|  | .68 | .53 |  | .65 | .50 | .32 |
|  | .59 | .65 |  | .40 | .44 | .64 |
|  | .37 | .86 |  | .25 | .28 | .86 |

*Note: λ* = standardized factor loading; *λ_RRB_* = standardized factor loading onto general restricted and repetitive behavior factor; *ε* = residual variance; *ω* = McDonald’s omega.

1. There were original 249 participants, although 43 were removed due to self-reporting invalid data, completing the questionnaire quickly, i.e., less than 500 seconds, or not wanting their data to be used. [↑](#footnote-ref-1)
